# PlzD modifies *Vibrio vulnificus* foraging behavior and virulence in response to elevated c-di-GMP

**DOI:** 10.1128/mbio.01536-23

**Published:** 2023-10-06

**Authors:** Tianyi Chen, Meng Pu, Sundharraman Subramanian, Dan Kearns, Dean Rowe-Magnus

**Affiliations:** 1 Department of Biology, Indiana University Bloomington, Bloomington, Indiana, USA; 2 Division of Gastroenterology and Hepatology, Mayo Clinic, Rochester, Minnesota, USA; 3 Department of Biochemistry and Molecular Biology, Michigan State University, East Lansing, Michigan, USA; 4 Department of Molecular and Cellular Biochemistry, Indiana University Bloomington, Bloomington, Indiana, USA; University of Hawaii at Manoa, Honolulu, Hawaii, USA; Michigan State University, East Lansing, Michigan, USA

**Keywords:** c-di-GMP, biofilms, *Vibrio vulnificus*, flagellar motility

## Abstract

Some marine bacteria can swim at speeds exceeding 50× their body length per second as they actively seek out fleeting microscale nutrient patches. Yet, even in vast marine environments, most micro-organisms live in surface-associated biofilm communities. Such astonishing speeds can pose a serious challenge to initiating biofilm formation, and our understanding of the mechanisms that regulate deceleration is lacking. Calcium, a major constituent of marine ecosystems, promotes biofilm formation in the marine bacterium and opportunistic human pathogen *Vibrio vulnificus* by increasing cellular levels of the key secondary signaling molecule bis-(3´-5´)-cyclic dimeric guanosine monophosphate (c-di-GMP). Here, we show that elevated c-di-GMP levels inhibit *V. vulnificus* swimming motility and identify a PilZ domain protein, PlzD, that mediates this effect. PlzD is localized at the flagellar pole in slow-moving, initially adherent, and mature biofilm cells. This positioning was partly dependent on the flagellar stator protein PomA and was regulated by c-di-GMP. Mutating the conserved RXXXR c-di-GMP binding motif of the PilZ domain abrogated PlzD activity. Single-cell tracking of bacterial swimming trajectories revealed that PlzD altered the foraging behavior of cells by slowing swimming speed and by decreasing the number of directional changes, ultimately limiting exploration of the surrounding 3D space. The cumulative effects were increased biofilm formation, aggregation, oyster colonization, and attenuated virulence in mice, phenotypes underpinning the evolution of *V. vulnificus* as a resilient environmental organism and potent human pathogen.

**IMPORTANCE:**

Many free-swimming bacteria propel themselves through liquid using rotary flagella, and mounting evidence suggests that the inhibition of flagellar rotation initiates biofilm formation, a sessile lifestyle that is a nearly universal surface colonization paradigm in bacteria. In general, motility and biofilm formation are inversely regulated by the intracellular second messenger bis-(3´-5´)-cyclic dimeric guanosine monophosphate (c-di-GMP). Here, we identify a protein, PlzD, bearing a conserved c-di-GMP binding PilZ domain that localizes to the flagellar pole in a c-di-GMP-dependent manner and alters the foraging behavior, biofilm, and virulence characteristics of the opportunistic human pathogen, *Vibrio vulnificus*. Our data suggest that PlzD interacts with components of the flagellar stator to decrease bacterial swimming speed and changes in swimming direction, and these activities are enhanced when cellular c-di-GMP levels are elevated. These results reveal a physical link between a second messenger (c-di-GMP) and an effector (PlzD) that promotes transition from a motile to a sessile state in *V. vulnificus*.

## INTRODUCTION

Biofilm formation, where micro-organisms live in surface-associated communities that are sheathed in a protective matrix of extracellular polysaccharides (EPS), proteins, and DNA (1, 2), is an ancient and universal survival mechanism that increases access to nutritional resources, provides protection from environmental insults and predation, and underscores environmental persistence (3, 4), symbiosis (5), lateral gene transfer (6), and the establishment of chronic infections (4).

The secondary signaling molecule bis-(3´-5´)-cyclic dimeric guanosine monophosphate (c-di-GMP) is a key regulator of biofilm formation (7 – 9). It is synthesized by diguanylate cyclases (DGC) bearing a Gly–Gly–Asp–Glu–Phe (GGDEF) domain and degraded by phosphodiesterases (PDEs) containing Glu–Ala–Leu (EAL) or His–Asp/Gly–Tyr-Pro (HD-GYP) domains (10, 11). Increases in c-di-GMP concentration generally repress flagellar-mediated swimming motility and virulence gene expression, while enhancing EPS production and biofilm formation (10, 12). A variety of effectors that bind c-di-GMP and elicit a physiological response have been identified (13). These include proteins containing degenerate GGDEF and EAL domains (14), the PilZ domain (15), the GGDEF-I-site-like (GIL) domain (16), motifs [e.g., I-site, Walker A, and w[F/L/M][T/S]R (17 – 22)] and RNA riboswitches (23 – 25). These binding sites can be found alone (26, 27) or coupled to output domains, the function of which is regulated by signal binding (28 – 31).

The first class of c-di-GMP protein-binding sites identified was the PilZ domain, which binds c-di-GMP via conserved RxxxR and (D/N)x(S/A)xxG motifs (30, 32). The paradigm for PilZ:c-di-GMP function is the post-translational regulation of swimming motility by YcgR of the enteric bacteria, *Escherichia coli* (33). *E. coli* swim through liquid environments by rotating multiple flagella, which have three major segments: the basal body, the hook, and the filament (34). The flagellar motor is part of the basal body and consists of a membrane-embedded stator and the cytoplasmic rotor (35). The stator, formed from the membrane proteins MotA and MotB, conducts protons. The rotor, comprised of FliG, FliM, and FliN, is a complex that exerts clockwise (CW)/counterclockwise (CCW) directional control of motor rotation. As protons travel through the stator, they generate torque at the stator-rotor interface to drive motor rotation (36). Electrostatic interactions between conserved charged residues in MotA and FliG are essential for coupling proton flow to mechanical motion (37, 38). When all the motors rotate CCW simultaneously, the individual flagellar filaments organize into a bundle and the cell is propelled forward in a straight run. Runs are interrupted by tumbles, short episodes of CW flagellar rotation that cause bundle dispersal and random re-orientation of the cell. The switch to CW flagellar rotation is regulated by reversible phosphorylation of the switch protein, CheY; phosphorylated CheY binds to the flagellar motor and increases the probability of CW motor rotation (39 – 42). Deletion of *yhjH*, which codes for a PDE, increases cellular c-di-GMP levels and inhibits *E. coli* swimming motility, whereas deletion of *ycgR* alone does not. However, loss of *ycgR* suppresses the motility defect of *yhjH* deletion, indicating that YcgR inhibits motility under conditions of elevated cellular c-di-GMP (28, 43). It has since been demonstrated that YcgR is an effector that binds c-di-GMP via its PilZ domain (44) and then interacts with the flagellar motor to slow motility and skew motor bias in the CCW direction (33, 44 – 46).

Marine environments occupy nearly 70% of the earth’s surface (47) and pose very different survival challenges compared to the nutrient-dense habitats of enteric bacteria. Yet, even in these vast ecosystems, most micro-organisms live in biofilms because, sustenance challenges notwithstanding, ambient conditions are highly dynamic and potentially harmful (48, 49). Understanding how marine bacteria decelerate to initiate biofilm formation is critical for understanding processes such as food-chain contamination (50, 51), the generation of microplastics that contaminate drinking water (52, 53), biocorrosion (54), the formation of artificial reefs (55), and genetic exchange (51, 56). The broad strokes of biofilm formation—slowing motility and approaching a surface, loosely associating with it (reversible attachment), and sticking tenaciously (irreversible attachment)—have been largely deciphered. However, many of the finer details, particularly for highly motile marine bacteria, remain largely unknown. What are the primary environmental cues that signal cells to slow down? How are they perceived and converted to secondary signals inside the cell that alter swimming behavior? What encourages cells to linger at a surface long enough to initiate biofilm formation instead of changing direction and moving away?

Some 90% of motile marine bacteria have a single polar flagellum (57) and some species can swim at astonishing speeds of >50× their body length per second (58 – 60) in search of evanescent microscale nutrient plumes (61, 62). *Vibrio* species are Gram-negative, halophilic, curved bacteria that are ubiquitous in estuarine and marine environments (63, 64). They have a single polar, sodium-driven flagellum (57) and the stator is composed of the MotA and MotB homologs, PomA and PomB. The flagellar motor of *Vibrio alginolyticus* can spin at speeds of 1,700 revolutions per second (58), far faster than the 300 revolutions per second observed for the motors of *E. coli* and *Salmonella typhimurium* (65, 66). *Vibrio* species swim using a distinct, three-step pattern that cycles between forward runs, reversals of ~180°, and flicks (run-reverse-flicks) that re-orient the cell (59, 67, 68). CCW rotation of the flagella pushes the cell forward (run), while CW rotation pulls it backward (reverse). Flicks occur during the transition from backward to forward swimming and re-orients the cell head ~90°. This strategy is preferred in marine environments where searching is unproductive and exploitation of an existing, albeit transient, resource takes precedence because bacteria can retrace their steps in the surrounding water and quickly relocate nutrients before they are dissipated by turbulent conditions (62, 69, 70). However, high speeds and acute flicks that may re-orient the cell away from a surface pose serious challenges to initiating biofilm formation.

A dozen *Vibrio* species including *V. vulnificus*, *V. cholerae,* and *V. parahaemolyticus* are pathogenic to humans (64, 71). They are autochthonous to estuaries and warm coastal waters, and their environmental persistence and transmission are bolstered by their ability to form biofilms on biotic and abiotic marine surfaces (72, 73). c-di-GMP is proposed to be a key regulator of *Vibrio* physiology, the genomes of which can code for more than 100 proteins predicted to make, break, or bind the signaling molecule (74). The *Vibrio* effector and YcgR homolog, PlzD, has been studied in some detail but its mechanism of action is unclear. As observed for *ycgR*, deletion of the *plzD* had little impact on *V. cholerae* swimming motility, while its over-expression inhibited motility and decreased intestinal colonization (75). PlzD was shown to tightly bind c-di-GMP with a dissociation constant (K_D_) of ~300 nM (32, 75). Both the apo (76) and holo structures of PlzD have been solved (32). Although both are dimeric, each monomer in the holo structure binds a single molecule of c-di-GMP, resulting in a major conformational change, whereby the C-terminal PilZ domain is rotated 123° toward the N-terminal YcgR domain with c-di-GMP sandwiched in the junction between them. Yet, inhibition of swimming motility by PlzD was reported to be independent of c-di-GMP binding since a mutant that was unable to bind c-di-GMP still suppressed motility when over-expressed (75). It is difficult to imagine that the activity of PlzD is unphased by such a dramatic structural reconfiguration upon c-di-GMP binding and that its function is indeed c-di-GMP independent. Moreover, the mechanism by which PlzD ultimately inhibits motility (i.e., subcellular localization, potential interacting partners, etc) has not been resolved. Similarly, deletion of *plzD* did not affect *V. alginolyticus* swimming motility, while its over-expression reduced motility (77). Again, this activity was reported to be independent of c-di-GMP binding since mutants bearing substitutions in the conserved ligand-binding pocket also inhibited motility when over-expressed. And despite PlzD localizing to the flagellar pole, its interaction with the flagellar motor was deemed too weak to inhibit motility (77), making its mechanism of action even more nebulous.

*V. vulnificus* naturally colonizes marine foodstuffs such as oysters, fish, and eel (78). Unlike other family members, which typically cause gastroenteritis, *V. vulnificus* is best known for causing devastating wound and septicemic infections (78, 79). The fatality rate of septicemic patients can exceed 50%, and *V. vulnificus* carries the highest death rate and per case economic burden (>$3.3 M) of any foodborne disease agent (80). We recently showed that calcium, a major constituent of marine ecosystems, triggers *V. vulnificus* biofilm formation by increasing cellular levels of c-di-GMP (81). Elevated levels of c-di-GMP induce expression of the biofilm-promoting EPS encoded by the *brp* locus (82 – 85) and the matrix protein, CabA (85 – 87). Here, we investigate the mechanism by which elevated cellular c-di-GMP levels inhibit *V. vulnificus* swimming motility to encourage biofilm formation. We show that PlzD inhibits swimming motility in response to elevated c-di-GMP levels, and mutation of the RXXXR motif suppressed this phenotype. PlzD localized to the flagellar pole in slow-moving, adherent, and biofilm cells in a c-di-GMP- and PomA-dependent manner. Single-cell tracking of bacterial swimming trajectories revealed that PlzD modified the foraging behavior of cells by slowing swimming speed and decreasing the number of directional changes to limit exploration of the surrounding 3D space. The cumulative effects were increased biofilm formation, aggregation and oyster colonization, and attenuated virulence in mice, phenotypes that bolster the evolution of *V. vulnificus* as a resilient environmental organism and potent human pathogen.

## RESULTS

### PlzD mediates inhibition of *V. vulnificus* swimming motility by elevated cellular c-di-GMP

**Fig 1:**
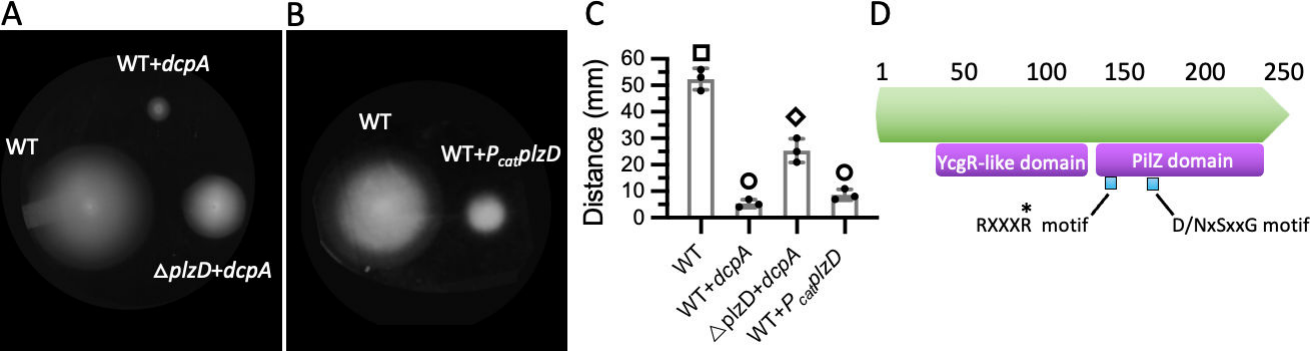
Inhibition of *V. vulnificus* motility by elevated cellular c-di-GMP is PlzD dependent. (A) Motility of WT control cells carrying the empty vector or expressing the DGC, DcpA. Also shown is the motility of the △*plzD* strain expressing *dcpA*. (B) Motility of the WT strain constitutively expressing *plzD* (*P_cat_plzD*). (C) A plot of the corresponding motility zones for the strains in A and B. It shows the respective mean values for each strain and error bars represent the standard deviation of triplicate assays. Different symbols above each bar indicate statistically significant differences between strains (one-way analysis of variance with Tukey’s multiple comparisons *post hoc* test, *P* < 0.001). Identical symbols indicate no significant difference between those specific strains. (D) domain structure of PlzD (green arrow), which contains an N-terminal YcgR and C-terminal PilZ domain (purple regions). The conserved RXXXR and D/NxSxxG motifs (blue) that comprise the c-di-GMP binding site are also shown. R^140^, which is part of the RXXXR motif, is marked with an asterisk.

Elevated cellular c-di-GMP levels promote *V. vulnificus* biofilm formation by increasing the production of EPS and the CabA matrix protein (82 – 87). To determine if elevated cellular c-di-GMP levels also impacted *V. vulnificus* swimming motility, colony spread by the wild-type (WT) strain with unaltered and elevated cellular c-di-GMP levels was assessed. Control cells harboring the empty expression vector were motile when inoculated into semi-solid agar (Fig. 1A), whereas colony spread was strongly inhibited in cells over-expressing *dcpA*, which codes for a *V. vulnificus* DGC that synthesizes c-di-GMP (88). A transposon screen revealed that disruption of *aot11_00285* partially relieved c-di-GMP-mediated inhibition of swimming motility. The protein encoded by *aot11_00285* shared 55% identity with PlzD of *V. cholerae*, including an N-terminal YcgR domain and a C-terminal PilZ domain bearing the highly conserved RxxxR and D/NxSxxG motifs that coordinate the binding of c-di-GMP (Fig. 1D). Consequently, *aot11_00285* was named *plzD*. The motility of an in-frame *plzD* deletion mutant expressing *dcpA* phenocopied that of the transposon mutant (Fig. 1A), and this could be complemented by ectopic expression of the wild-type gene (Fig. S1A). Although deletion of *plzD* had little effect on swimming motility when c-di-GMP levels were unaltered (Fig. S1B), its constitutive expression strongly inhibited motility (Fig. 1B and C). These results suggested that the inhibition of motility by elevated cellular c-di-GMP levels was linked in part to the activity of PlzD.

### Mutation of the PilZ domain inhibits PlzD function

**Fig 2:**
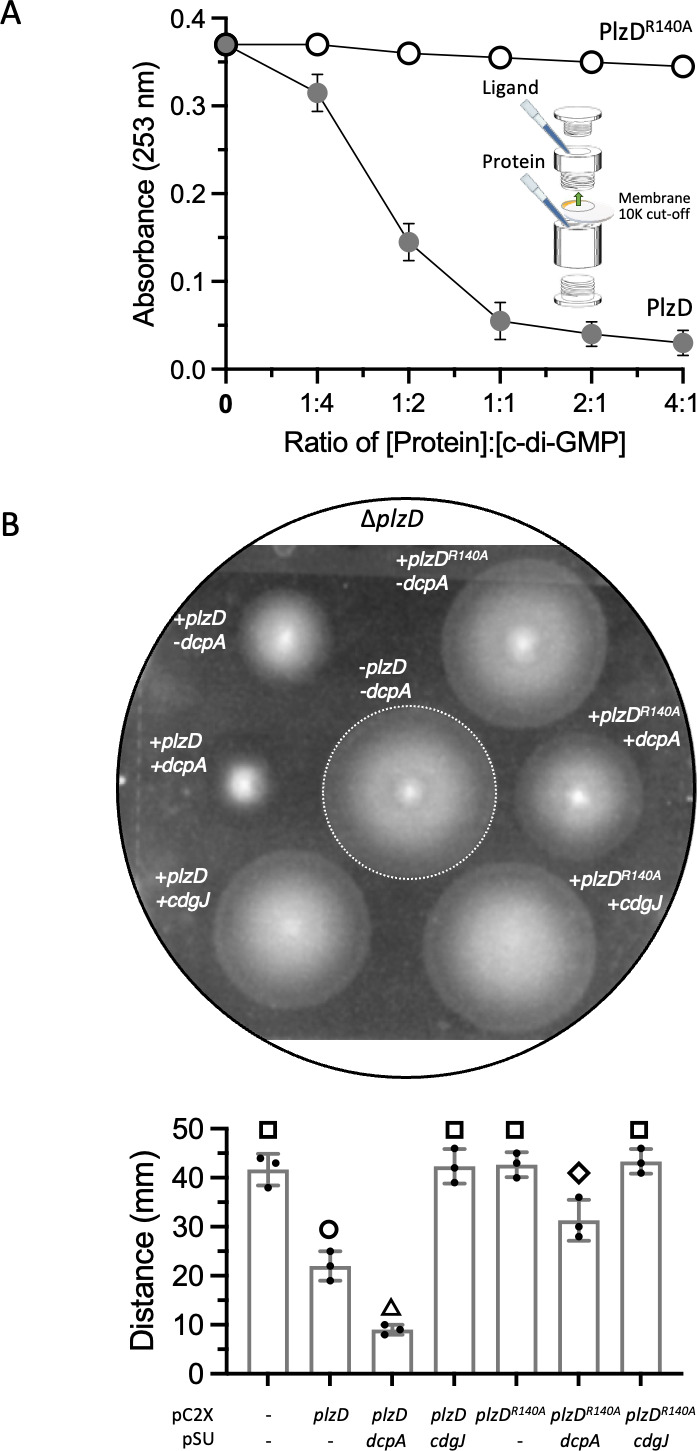
Mutation of the RXXXR motif abrogates c-di-GMP binding and PlzD function. (A) Plot showing the binding of c-di-GMP by purified wild-type PlzD and PlzD^R140A^. The concentration of unbound c-di-GMP remaining in the ligand chamber following micro-equilibrium dialysis (inset) was determined by measuring the absorbance at 253 nm and plotted versus the amount of protein added to the opposing chamber (the protein:ligand ratio). Points represent the mean of three independent assays and error bars represent the standard deviation. (B) Motility of the ∆*plzD* mutant expressing the indicated gene(s). Plasmids are as follows: pC2X, the empty vector for expression of *plzD* or *plzD^R140^*; pSU, the empty vector for expression of *dcpA* or *cdgJ*. The control strain is outlined with a dotted white line. The plot below shows the respective mean values for each strain and error bars represent the standard deviation of triplicate assays. Different symbols above each bar indicate statistically significant differences between strains (one-way analysis of variance with Tukey’s multiple comparisons *post hoc* test, *P* < 0.01). Identical symbols indicate no significant difference between those specific strains.

*V. cholerae* PlzD tightly binds c-di-GMP (32, 75) and we sought to determine if *V. vulnificus* PlzD also bound the signaling molecule. Structural modeling suggested that the predicted c-di-GMP binding site of *V. vulnificus* PlzD closely aligned with the known c-di-GMP binding site of PlzD from *V. cholerae* (Fig. S2A). Notably, the respective DXSXXG motifs mirrored one another. R^136^ and R^140^ of the RXXXR motif of *V. cholerae* PlzD engage in H-bonding and stacking interactions, respectively, with a guanine base. These roles were conserved in the RXXXR motif of *V. vulnificus* PlzD, with R^136^ base stacking and R^140^ H-bonding with the guanine base. The conservation of binding site stereochemistry suggested that *V. vulnificus* PlzD activity might also be dependent on interaction with c-di-GMP. Although wild-type *V. cholerae* PlzD binds c-di-GMP, a PlzD^R140A^ mutant does not (32, 75). The analogous amino acid change was introduced into *V. vulnificus* PlzD and the binding of c-di-GMP by the wild-type and mutant proteins was qualitatively measured by micro-equilibrium dialysis, where the concentration of a ligand is varied and binding is measured at equilibrium (89, 90). Both wild-type PlzD and PlzD^R140A^ were stably produced to comparable levels and purified (Fig. S2B). A device with two chambers separated by a membrane (10 kDa cut-off) was loaded with a fixed concentration of c-di-GMP in one chamber and varying concentrations of sample protein in the other (Fig. 2A, inset). The protein, being too large to cross the membrane, remains in its chamber. The ligand, however, can freely pass through the membrane to interact with the protein and, if so, is retained in the adjacent chamber. Hence, the concentration of c-di-GMP in the ligand chamber should remain constant following equilibration if the protein introduced into the adjacent chamber is unable to bind c-di-GMP. However, if the protein can stably bind c-di-GMP, then the concentration of c-di-GMP in the ligand chamber should decrease as the concentration of protein added to the adjacent chamber increases. A plot of the UV absorbance at 253 nm of samples taken from the ligand chamber versus the ligand:protein ratio yields qualitative information of the relative c-di-GMP binding affinity (91). Significant decreases in c-di-GMP concentration in the ligand chamber were observed following equilibration with increasing concentrations of PlzD, whereas the c-di-GMP concentration in the ligand chamber remained unchanged over the same range of PlzD^R140A^ concentrations (Fig. 2A). This suggested that PlzD was able to stably bind c-di-GMP while PlzD^R140A^ could not, and that mutation of R^140^ prevented ligand binding. Plotting the fraction of free c-di-GMP versus the concentration of protein yielded a K_D_ of 3.9 µM for PlzD (Fig. S2C).

The effect on motility of *plzD* and *plzD^R140A^* was assessed in a Δ*plzD* background under decreased [by co-expressing *cdgJ* of *V. cholerae*, which codes for a PDE that degrades c-di-GMP (92)], unaltered, and elevated (by co-expressing *dcpA*) cellular c-di-GMP levels. Control cells were motile (Fig. 2B, middle). Expression of *plzD* alone inhibited motility twofold, while expression of *plzD^R140A^* alone had no effect on motility (top left and right). Expression of *plzD* together with *dcpA* inhibited motility 3.5-fold (middle left). However, co-expression of *plzD^R140A^* and *dcpA* resulted in only a 1.6-fold inhibition (middle right), consistent with the inability of PlzD^R140A^ to mitigate the inhibitory effect of elevated c-di-GMP on motility. Motility was not inhibited when either *plzD* or *plzD^R140A^* was co-expressed with *cdgJ* (bottom left and right). Together, these results suggested that c-di-GMP-regulated inhibition of motility by PlzD was dependent on a functional PilZ domain and binding of c-di-GMP.

### PlzD alters the foraging behavior *V. vulnificus*

**Fig 3:**
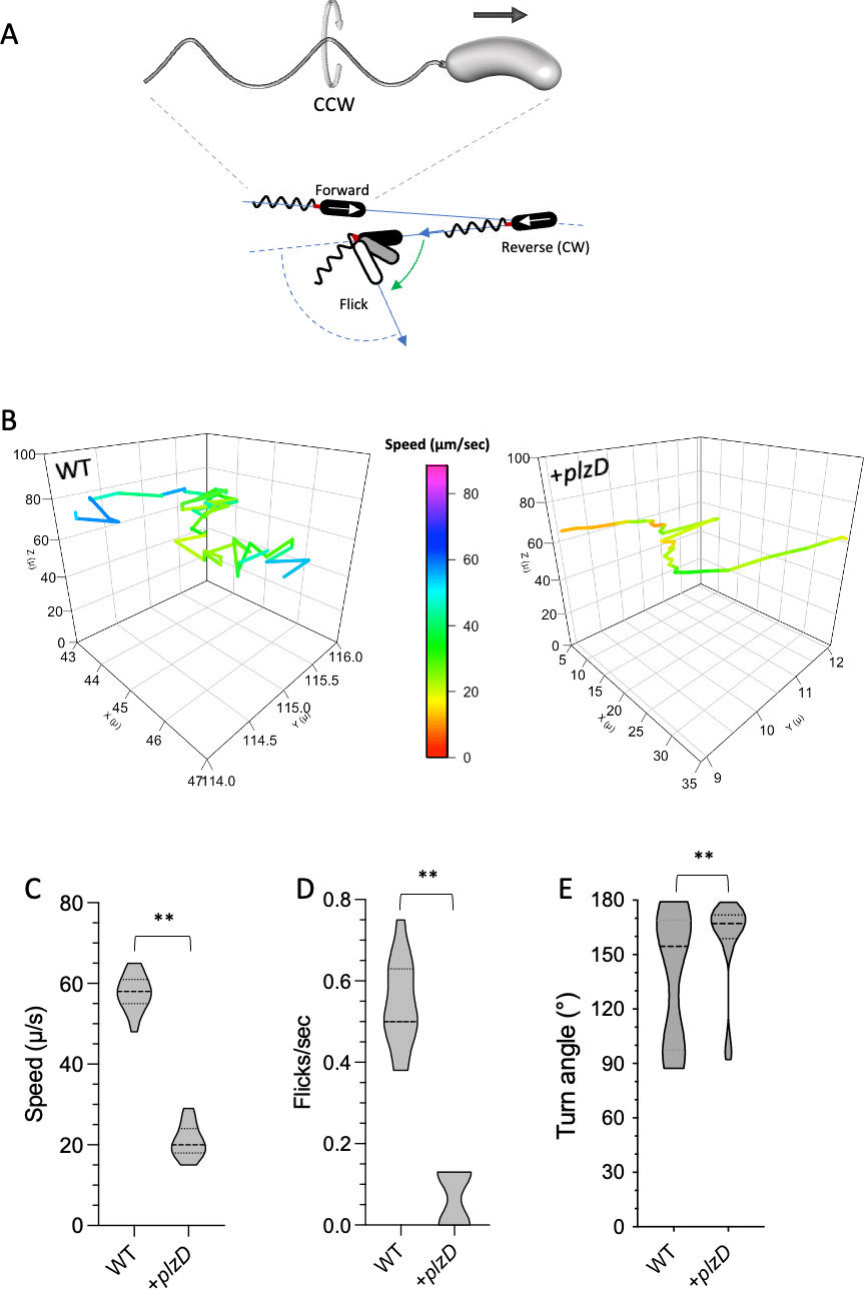
PlzD alters the swimming behavior of *V. vulnificus* cells. (A) Representation of a *Vibrio* cell propelled by a single polar flagella with a left-handed helical twist. The direction of flagellar rotation determines whether the cell is pushed (CCW rotation) or pulled (CW rotation). The forward-reverse-flick motility pattern of *Vibrio* species is depicted below. Switching from CCW to CW rotation results in near 180° reversals. Flicks result from buckling of the flagellar hook (red) on resuming forward movement. A smaller deflection correlates with a larger flick angle. (B) Reconstructed representative 3D swimming trajectories of WT cells carrying the empty vector or expressing *plzD* (+*plzD*). A colored speed scale is also shown. (C–E) Quantifying the swimming speed, number of flicks, and flick angles of swimming cells. Violin plots show the median, first and third quartiles, and data range for at least 100 tracks for each strain. Statistically significant differences between the samples were determined by an unpaired Student’s *t*-test (two-tailed) in C (**P < 0.001) and an unpaired Mann-Whitney test (two-tailed) in D and E (**P < 0.001).

To better characterize the effect of *plzD* expression on the run-reverse-flick swimming pattern of *V. vulnificus* (Fig. 3A), we tracked single-cell trajectories in 3D using high-speed, time-lapse phase contrast microscopy. Planktonic wild-type cells exhibited classic run-reverse-flick patterns of forward and reverse runs peppered with re-orientations (turns) in the x, y, and z planes that promoted extensive exploration of the surrounding space (Fig. 3B), with an average speed of 55 µm/s (Fig. 3C). An average of 0.5 flicks per second was observed for these cells (Fig. 3D) and although the turn angles (ϴ) ranged from 87° to 179°, they primarily fell into two clusters of ~93° (flicks) and 167° (reversals), respectively (Fig. 3E). Wild-type cells expressing *plzD* produced curvilinear traces and did not exceed 30 µm/s. Notably, the median turn angle was large (~168°) and the flick frequency was eightfold lower than wild-type cells (0.06 flicks per second). Consequently, cells tended to meander along only two axes with minimal backtracking. These results suggested that PlzD functioned to decrease cell swimming speed, decrease the number of directional changes in rotation, and decrease flick frequency, thereby limiting tri-axial movement and exploration of the surrounding 3D space.

### PlzD localization to the flagellar pole is c-di-GMP dependent

**Fig 4:**
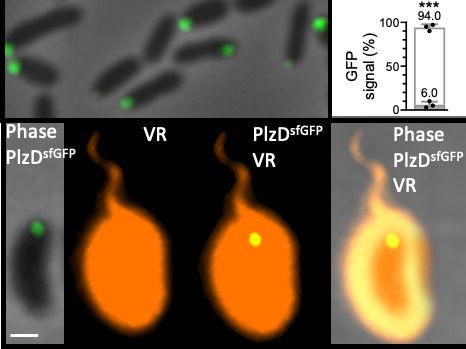
PlzD localizes to the flagellar pole. Top, phase/fluorescence overlay image (100× magnification) of ∆*plzD* cells expressing *dcpA* and *plzD^sfgfp^*. To the right is a plot showing the percentage of cells with a polar (white bar) or cytoplasmic (gray bar) fluorescent signal. Bars indicate the respective mean values (*n* = 100) for each strain and error bars represent the standard deviation of triplicate assays. Statistically significant differences (***P < 0.0001) were determined by an unpaired Mann-Whitney test (two-tailed). Bottom, representative overlay image of ∆*plzD* cells expressing *dcpA* and *plzD^sfgfp^* that were also stained with the lipophilic membrane stain, Vybrant Red (VR) to show the flagella. The scale bar is 1 µm.

**Fig 5:**
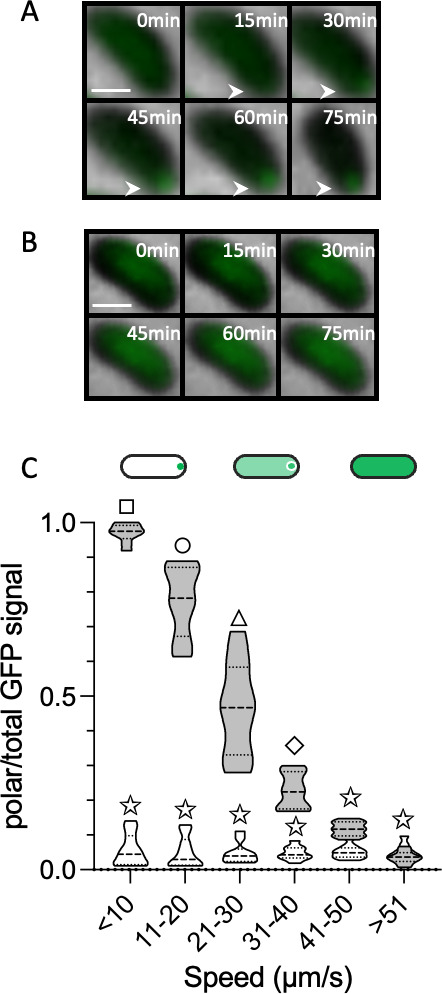
Polar localization of PlzD is c-di-GMP dependent. (A and B) Time-lapse overlay of phase and GFP fluorescence images (100× magnification) showing the subcellular distribution of PlzD^sfGFP^ and PlzD^R140AsfGFP^, respectively, in ∆*plzD* cells 0–75 min after cellular c-di-GMP levels were increased by inducing *dcpA* expression with L-arabinose. White arrows highlight polar localization of PlzD^sfGFP^. The scale bar in the upper left box is 1 µm. (C) Quantification of the polar PlzD^sfGFP^ (gray) and PlzD^R140AsfGFP^ (white) ratio of cells swimming at the indicated speeds. Violin plots show the median, upper and lower quartiles, and data range for at least 100 tracks for each strain. Different symbols above each bar indicate statistically significant differences between strains (one-way analysis of variance with Tukey’s multiple comparisons *post hoc* test, *P* < 0.01). Identical symbols indicate no significant difference between those specific strains.

Considering its effect on motility, we sought to determine the subcellular location of PlzD under elevated c-di-GMP levels (i.e., in cells co-expressing *dcpA*). Under elevated cellular c-di-GMP conditions, a polar fluorescent signal was observed in 94% of Δ*plzD* cells that expressed a construct coding for a stable and functional PlzD-superfolder green fluorescent protein (GFP) fusion (PlzD^sfGFP^; Fig. 4 and Fig. S3). When cells were also stained with Vybrant DiD (a fluorescent lipophilic dye that labels bacterial membranes), both cell bodies and the sheathed flagella were uniformly labeled by the dye, and PlzD^sfGFP^ clearly localized to the flagellar pole. This suggested that PlzD might interact with the flagellar complex to slow motility. Since mutation of the PilZ domain mitigated its ability to inhibit motility, we determined if c-di-GMP affected its subcellular location. Time-lapse microscopy was used to monitor the subcellular localization of PlzD^sfGFP^ in Δ*plzD* cells in response to increasing c-di-GMP levels. When cells were seeded into microfluidic chambers in media that lacked arabinose (i.e., unaltered cellular c-di-GMP conditions), the PlzD^sfGFP^ signal was diffuse (Fig. 5A). The media was then exchanged to one that included arabinose to induce *dcpA* expression and increase the cellular c-di-GMP concentration. Under these conditions, the PlzD^sfGFP^ signal began to coalesce at one pole and was clearly visible 30 min post-induction. Conversely, PlzD^R140AsfGFP^ remained dispersed both before and after c-di-GMP levels were elevated (Fig. 5B). Further support for polar localization by PlzD was obtained by adding Ca^2+^ to the media, which is known to increase cellular c-di-GMP levels in *V. vulnificus* (81). The PlzD^sfGFP^ signal was diffuse in cells prior to the addition of Ca^2+^, but polar in the presence of Ca^2+^ (Fig. S4).

The movement of single cells within 30 µm of the coverslip surface was tracked by high-speed, time-lapse fluorescence microscopy and cells were binned according to speed. The ratio of polar to total PlzD^sfGFP^ signal was determined for each cell and plotted versus speed. A correlation emerged between cell speed and PlzD^sfGFP^ subcellular localization. The PlzD^sfGFP^ signal was completely dispersed in rapidly swimming cells but was almost entirely polar in slow-moving and stationary cells (Fig. 5C). Indeed, the ratio of polar PlzD^sfGFP^ signal increased as cell speed decreased. The greatest variability in PlzD^sfGFP^ location occurred at speeds between 20 µm/s and 30 µm/s. A polar PlzD^sfGFP^ signal was more commonly observed in cells swimming at speeds closer to 20 µm/s rather than those swimming at speeds near the upper end of the bin. Conversely, the signal for PlzD^R140AsfGFP^ remained dispersed at all speeds. Collectively, these results suggested that PlzD localization to the flagellar polar was regulated by c-di-GMP and required a functional RXXXD motif.

### Localization of PlzD to the flagellar pole is dependent on PomA

**Fig 6:**
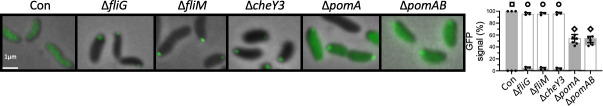
PlzD localization to the flagellar pole is dependent on PomA. Localization of GFP (control) or PlzD^sfGFP^ in *∆plzD* cells that also carry deletions of the indicated rotor (*fliG* and *fliM*), stator (*pomA* and *pomB*), and switch (*cheY*) genes. Images were taken at 100× magnification and the scale bar is 1 µm. On the right is a plot showing the percentage of cytoplasmic (gray bar) and polar (white bar) PlzD^sfGFP^ signal in cells (*n* = 100) from each background. Bars indicate the respective mean values for each strain and error bars represent the standard deviation of triplicate assays. Different symbols above each bar indicate statistically significant differences between strains (one-way analysis of variance with Tukey’s multiple comparisons *post hoc* test, *P* < 0.001). Identical symbols indicate no significant difference between those specific strains.

Localization of PlzD to the flagellar pole suggested that it interacted with the flagellar complex to slow motility. To identify potential interacting partners of PlzD at the flagellar pole, ∆*plzD* strains harboring deletions in genes coding for rotor (*fliG* and *fliM*), stator (*pomA* and *pomB*), and switch (*cheY3*) components were generated, and PlzD^sfGFP^ was then localized in each background under elevated cellular c-di-GMP conditions. The fluorescent signal in control cells expressing *^sf^gfp* alone was entirely diffuse (Fig. 6). Nearly all (>95%) of the PlzD^sfGFP^ signals were polar in the ∆*plzD*∆*fliG*, ∆*plzD*∆*fliM*, and ∆*plzD*∆*CheY3* backgrounds. However, the PlzD^sfGFP^ signal was partially diffuse in the ∆*plzD*∆*pomA* and ∆*plzD*∆*pomAB* backgrounds (55% and 45% polar, respectively). These results suggested that the localization of PlzD to the flagellar pole was partly dependent on PomA, but on FliG, FilM, or CheY3.

### PlzD promotes biofilm formation, aggregation, and oyster colonization

**Fig 7:**
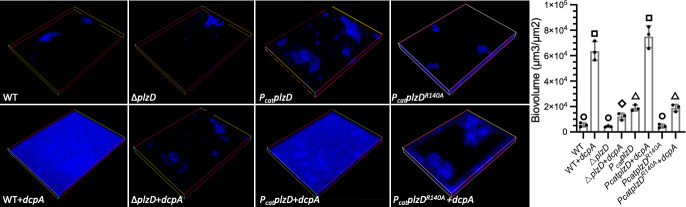
PlzD promotes biofilm formation. Hoechst-stained fluorescent images (40× magnification) of biofilms formed under constant flow by WT, ∆*plzD,* or constitutive expression strains (*P_cat_plzD* and *P_cat_plzD^R140A^*) under unaltered and elevated (+*dcpA*) cellular c-di-GMP conditions. A plot of the corresponding biofilm biovolumes is to the right of the panels. The plot shows the mean values for each strain and error bars represent the standard deviation of triplicate assays. Different symbols above each bar indicate statistically significant differences between strains (one-way analysis of variance with Tukey’s multiple comparisons *post hoc* test, *P* < 0.05). Identical symbols indicate no significant difference between those specific strains.

Given the overall effect of *plzD* expression on motility, we sought to determine its impact on surface colonization. Wild-type, Δ*plzD*, and strains that constitutively express either *plzD* or *plzD^R140A^* (*P_cat_plzD* and *P_cat_plzD^R140A^*) were separately seeded into microfluidic chambers under constant flow and biofilm development was monitored by fluorescence microscopy. The wild-type, *ΔplzD,* and *P_cat_plzD^R140A^* strains failed to produce a monolayer, and microcolony formation by these strains was scant (Fig. 7). Both monolayer and microcolony formation were superior for the *P_cat_plzD* strain and the overall biomass was sixfold higher by comparison. An extensive monolayer and robust microcolony formation were observed for the wild-type strain under elevated c-di-GMP conditions (expressing *dcpA*), and the biofilm biomass was nearly 11-fold higher than the parental strain. A similar phenotype was observed for the *P_cat_plzD* strain expressing *dcpA*, with a biomass increase of 13-fold relative to the wild-type strain. However, the biomass of the ∆*plzD* strain expressing *dcpA* was only twofold higher than wild type and monolayer formation remained poor. Although the biomass of the *P_cat_plzD^R140A^* strain expressing *dcpA* was only threefold higher than wild type, constitutive *plzD^R140A^* expression did encourage sparse monolayer formation, suggesting that PlzD^R140A^ retained some residual activity. These results suggested that PlzD functioned to enhance monolayer formation and subsequent biofilm development, and mutation of the RXXXR motif hinders PlzD function.

**Fig 8:**
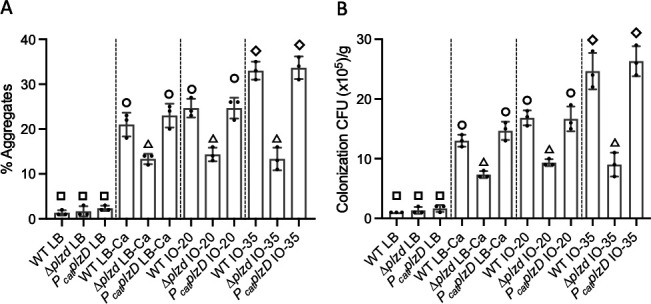
PlzD promotes *V. vulnificus* aggregation and oyster colonization. (A) Aggregation by WT, ∆*plzD,* or complemented (*P_cat_plzD*) strains grown in LB, LB containing calcium (LB-Ca), Instant Ocean at 20 ppt (IO-20) or 35 ppt (IO-35). Vertical dashed lines separate each media type. (B) Oyster colonization by the same strains in A. Oysters were inoculated and the number of CFU per gram of oyster meat was determined. Plots show the mean values for each strain and error bars represent the standard deviation of triplicate assays. Different symbols above each bar indicate statistically significant differences (one-way analysis of variance with Tukey’s multiple comparisons *post hoc* test, *P* < 0.01) between samples. Identical symbols indicate no significant difference between those specific strains.

Oysters size-select particles for ingestion. Those less than 5 µm in size are poorly retained (<20% efficiency), while particles greater than 5 µm are captured with nearly 100% efficiency (93). Since planktonic *Vibrio* cells are just 1 μm × 1 μm × 2 μm, self-association (aggregation) could boost their retention by feeding bivalves. To assess the role of *plzD* in aggregation, we first used fluorescence-activated cell sorting (FACS) analysis to determine the percentage of aggregates in 50 µm filtrates of wild-type, ∆*plzD,* and *P_cat_plzD* strains that were grown in Luria-Bertani (LB) (without or with added 15 mM Ca^2+^) and Instant Ocean (IO) at 20 ppt or 35 ppt. Each strain existed mainly as single cells (<1% aggregates) when grown in LB (Fig. 8A). The addition of Ca^2+^ to LB increased aggregation by each strain; however, deletion of *plzD* decreased aggregation by 35% relative to the wild-type and *P_cat_plzD* strains. A similar pattern was observed when the strains were grown in IO-20. The difference in aggregation between strains was greatest in IO-35, where aggregation by the ∆*plzD* strain was only 30% that of the wild-type and *P_cat_plzD* strains. To determine if the difference in aggregation translated to a difference in oyster colonization, the strains were inoculated into separate tanks and their ability to colonize oysters was determined by CFU plate count (Fig. 8B). There was a strong correlation between the level of aggregation and the extent of oyster colonization. A low level of oyster colonization (~1.6 × 10^5^ CFU/g body weight) was observed for all three strains when grown in LB. This increased significantly when the strains were grown in LB with Ca^2+^, but colonization by the ∆*plzD* strain was decreased by 36% relative to the wild-type and *P_cat_plzD* strains. A similar pattern was observed when the strains were grown in IO-20. Again, the difference in colonization was greater when the strains were grown in IO-35, where colonization by the wild-type and *P_cat_plzD* strains was 2.5× that of the ∆*plzD* strain. These results suggested that PlzD functions to promote biofilm formation, aggregation, and oyster colonization in estuarine and marine environments.

### Constitutive PlzD production attenuates *V. vulnificus* virulence

**Fig 9:**
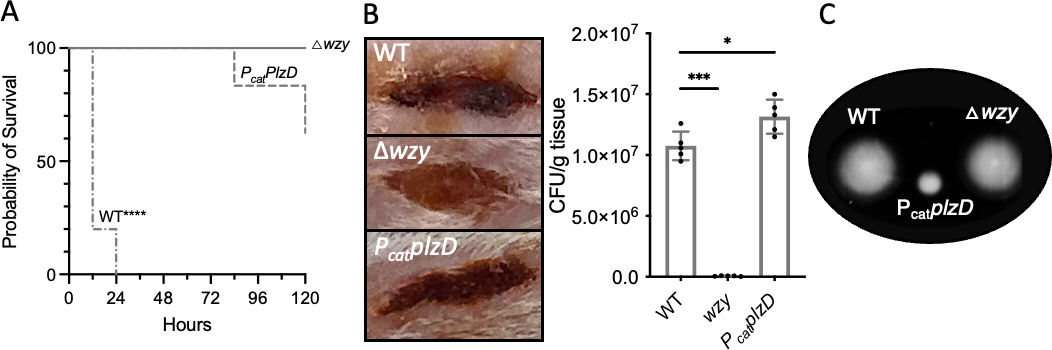
PlzD attenuates *V. vulnificus* virulence. (A) Survival curve for mice injected with the WT strain, a mutant strain that fails to produce capsule (∆*wzy*), or a strain that constitutively expresses *plzD*. Five-week-old female CD-1 mice housed under pathogen-free conditions were pretreated with an intraperitoneal injection of iron dextran (250 µg of iron-dextran per gram of body weight) 60 min before inoculation of excisional skin wounds with the bacterial strains. Groups of mice (*n* = 5) were injected intraperitoneally with 0.1 mL of 10^7^ CFU of each bacterial strain. Mice were observed for up to 120 h post-infection with the recording of deaths or moribund animals. Statistically significant differences between the WT strain and the ∆*wzy* and *plzD* strains were determined by the Log-rank (Mantel-Cox, *P*-value, ****<0.0001) test. (B) Representative images of excisional skin wounds 8 h post-infection with 10^7^ CFU of the same strains. To the right is a plot of the CFU recovered per gram of tissue from each wound site after 24 h. The plot shows the mean values for each strain and error bars represent the standard deviation of triplicate assays. Statistically significant differences from WT (one-way analysis of variance with Dunnett’s multiple comparisons *post hoc* test, *P*-values, *<0.05 and ***<0.001) are shown. (C) Motility of strains in A.

The effect of *plzD* expression on motility led us to investigate its impact on *V. vulnificus* virulence. Four strains were tested: wild type, ∆*plzD*, a ∆*wzy* mutant that fails to produce the anti-phagocytic capsular polysaccharide, and a strain that constitutively expressed *plzD* (*P_cat_plzD*). There was no difference in growth between the strains (Fig. S5). Groups of five mice were injected i.p. with 10^7^ CFU of each strain and survival was tracked over the course of 5 days. All mice infected with the wild-type strain died within 24 h (Fig. 9A), and tissue necrosis and hemorrhaging were observed in the vicinity of the wound site (Fig. 9B). Results with the ∆*plzD* strain were indistinguishable from the wild-type strain (Fig. S6). Conversely, all mice infected with the ∆*wzy* strain survived and the tissue at the wound site appeared healthy. Although two of the mice infected with the strain that constitutively expressed *plzD* succumbed, the first death did not occur until 84 h post-infection and three subjects survived the full 5 days. Bacteria were re-isolated from the blood of these two mice and the integrated *P_cat_plzD* region from 20 random clones (10 from each mouse) was amplified and sequenced. Nonsense mutations (8T > G, 19A > T, 68T > G, 423C > G) within *plzD* were identified in 19 of the clones and another bore a G > C mutation within the ribosome binding site (RBS) of *P_cat_* that likely inhibited translation initiation. This suggested that these animals succumbed to *V. vulnificus* infection due to the emergence of suppressor mutants that no longer constitutively expressed *plzD*. Finally, the wounds of mice that were exposed to the strain that constitutively expressed *plzD* exhibited signs of necrosis but there was very little bleeding in the surrounding tissue. The *P_cat_plzD* strain was also far less motile than the parental and Δ*wzy* strains (9C). Collectively, these results suggested that constitutive *plzD* expression attenuated *V. vulnificus* virulence in mice by impeding dissemination from the site of infection.

## DISCUSSION

Motile bacteria cannot “coast” in aqueous environments and must keep rotating their flagella to swim (94). The rotational speed of the Na^+^-driven flagellar motor for some *Vibrio* species has been reported to be as high as 1,700 revolutions per second (58), making these among the fastest molecular motors in biological systems. Slowing such astonishing motor speeds is essential when switching from a motile to sessile lifestyle. There are several post-translational mechanisms by which rapid deceleration of the Na^+^-driven flagellar motor could be achieved, such as through a reduction in [Na^+^] that could occur in estuaries where fresh and saltwater mix (95), by a transient drop in cellular ATP concentrations (96), or by the activity of proteins that function as a brake to halt rotation of the flagellar motor (33) or as a clutch that uncouples rotation of the flagellar motor from the flagellar filament (97).

The paradigm for c-di-GMP-mediated, post-translational control of swimming motility is YcgR (33). In response to elevated c-di-GMP concentrations, YcgR binds c-di-GMP and interacts with the flagellar motor to suppress motility (33, 98). c-di-GMP binding triggers rearrangement of the PilZ domain and the formation of a MotA-binding patch comprised of the RXXXR motif and the C-tail helix α3 of the PilZ domain (44). Monomeric YcgR:c-di-GMP is reported to stably bind at the stator-rotor interface, interacting with MotA via its C-terminal PilZ domain and FliG via its N-teminal YcgR domain, to disrupt rotor-stator interactions (44, 99). These interactions initially alter motor bias and then decrease motor speed (46).

The mechanism of action for YcgR homologs in *Vibrio* species is not as refined. *V. cholerae* PlzD tightly binds c-di-GMP and undergoes a large conformational change (32, 75), yet, the activity of PlzD was reported to be c-di-GMP independent since both wild-type and c-di-GMP binding mutants of PlzD inhibited swimming motility when over-expressed to high levels (75). Moreover, PlzD was required for intestinal colonization but not biofilm formation. The function of PlzD in *V. alginolyticus* is even murkier. As in *V. cholerae*, both wild-type and c-di-GMP binding mutants of PlzD inhibited *V. alginolyticus* swimming motility when highly over-expressed, and although PlzD localized to the flagellar pole, its interaction with the flagellar motor was concluded to be too weak to inhibit rotation (77). PlzD aggregation was suggested as the cause of motility inhibition; this was most likely due to high-level *plzD* expression under low cellular c-di-GMP conditions.

In this study, *plzD* was recovered from a transposon mutant screen to identify insertions that relieved c-di-GMP-mediated inhibition of motility in *V. vulnificus*. The function of *plzD* was then examined under low-level inducing conditions partnered with varied cellular c-di-GMP levels to better define its role and the requirement for c-di-GMP binding for activity. PlzD bears the conserved RXXXR and D/NxSxxG motifs that coordinate c-di-GMP binding, and its predicted structure suggested that PlzD could bind c-di-GMP. Equilibrium dialysis was used to monitor interactions between PlzD and c-di-GMP by measuring changes in the UV absorbance of c-di-GMP (91). Although UV measurements are not as sensitive as radioligand detection (32), this approach still permitted qualitative measurement of relative binding by wild-type and mutant PlzD. Wild-type PlzD was clearly able to bind c-di-GMP, whereas PlzD^R140A^ did not. The K_D_ of 3.9 µM for PlzD was higher than reported for *V. cholerae* PlzD (32, 75) and YcgR (28) using radioligand; however, it was on par with the K_D_ of other c-di-GMP effectors measured using this technique (91). The expression of wild-type *plzD* did not impact swimming motility when cellular c-di-GMP levels were depleted, but it mediated motility inhibition when c-di-GMP levels were left unaltered or were elevated. Conversely, the RXXXR mutant had no impact on swimming motility, regardless of the cellular c-di-GMP level. Since mutating the RXXXR motif or decreasing cellular c-di-GMP levels suppressed PlzD-mediated inhibition of motility, we propose that PlzD not only binds c-di-GMP, but that ligand binding regulates its activity.

Our data suggest that PlzD modifies *V. vulnificus* swimming behavior in two ways. The first is to decrease swimming speed. PlzD localized to the flagellar pole in a c-di-GMP- and PomA-dependent manner, and polar localization was most often observed in slow-moving, adherent, and biofilm cells. Tracking of single-cell trajectories revealed that *plzD* expression significantly reduced the speed of swimming cells (from more than 60 µm/s to below 30 µm/s). This is notable since flicking by *Vibrio* species correlates with swimming velocity, with a threshold of ~20–30 µm/s being required to induce a sufficient compressive load for flicking to occur (67, 68). Consequently, the probability of swimming cells to re-orient away from a surface that is being probed for potential colonization decreases as cells decelerate. Interactions between PlzD and PomA at the flagellar pole could perturb rotor-stator contacts and slow swimming speed, as reported for YcgR (44, 99). The second is to decrease switches between the forward and reverse swimming directions. The rapid, exploratory swim tracks of wild-type cells were peppered with reversals, flicks, and backtracks, while cells expressing *plzD* produced slow-moving, meandering, curvilinear traces with few flicks and backtracks. Although polar PlzD localization was not dependent on rotor proteins, this does not preclude transient interactions between them that could regulate CW/CCW rotational control, as proposed for YcgR, the N-terminus of which interacts with rotor proteins to regulate motor switching (44). It is also conceivable that PlzD instead interacts with known or novel components of the chemotaxis pathway to bias motor switching (100, 101). Additional studies are needed to ascertain if PlzD alters motor speed and bias concurrently or consecutively, if interactions with motor proteins in addition to PomA are required, and if PlzD function is mediated by non-motor (e.g., chemotaxis) proteins.

The phenotypic effects of *plzD* expression included increased biofilm formation, self-aggregation, and oyster colonization, important survival and colonization phenotypes for *V. vulnificus*. We propose that the restricted exploratory range of cells expressing PlzD increases linger time in the vicinity of a surface to promote initial attachment (102). PlzD expression also attenuated virulence in mice, most likely by impeding dissemination from the site of infection (103). Unlike YcgR, the loss of PlzD did not entirely prevent c-di-GMP-mediated inhibition of motility (46). Hence, additional mechanisms exist to slow motility in response to elevated c-di-GMP levels. Previous studies of five PilZ domain proteins in *V. cholerae* demonstrated overlap in their function: PlzB, PlzC, and PlzD regulated intestinal colonization and motility, and PlzB and PlzC were positive regulators of biofilm formation (75). Similar overlap may exist in *V. vulnificus*. Studies are under way to identify additional c-di-GMP-dependent systems of motility control that co-ordinate with the activity of PlzD.

## MATERIALS AND METHODS

### Strains and growth conditions

*V. vulnificus* strain ATCC27562 was used. *E. coli* S17.1λπ was used for cloning, plasmid maintenance, and conjugation to *V. vulnificus*. LB was purchased from BD Difco. The following antibiotics and additives were purchased from Sigma and used at the indicated concentrations: ampicillin (Ap) at 100 µg/mL, colistin (Col) at 12.5 µg/mL, tetracycline (Tc) at 15 µg/mL, chloramphenicol (Cm) at 2 µg/mL for *V. vulnificus* and 25 µg/mL for *E. coli*, kanamycin (Kn) at 160 µg/mL for *V. vulnificus* and 25 µg/mL for *E. coli*, trimethoprim (Tp) at 10 µg/mL, gentamicin (Gm) at 35 µg/mL, and isopropyl-β-D-thiogalactopyranoside (IPTG) at 10 µM or 100 µM. Instant Ocean was used at 20 ppt (IO-20) or 35 ppt (IO-35) and peptone was added to 1% where indicated.

### Transposon mutagenesis

A transposon mutagenesis library of wild-type cells expressing *dcpA* from pSU38GT (83) was generated by conjugation with *E. coli* S17.1λπ that carried the mini-Tn10 delivery vector pNKTXI-SceI (104). Transconjugants were selected on plates containing LB medium with Col, Kn, and Gm. A total of 12,530 mutants arrayed in 384-well plates and grown with shaking at 30°C overnight before 2 µL of each mutant was inoculated into 245 × 245 mm plates (Corning) containing 200 mL of motility medium (LB with 0.3% agar). The plates were incubated for 12 h at 30°C and mutants exhibiting increased motility relative to wild-type controls were manually picked, grown overnight in 96-well plates containing fresh medium, and rescreened in motility medium to confirm the phenotype. The *P_bad_dcpA* promoter region of each motility mutant from this second round of screening was verified as unaltered by sequencing (Eurofins Genomics). Mutants were grown in fresh LB Col Kn Gm medium to an optical density (OD_600_) of 1.0, and 50 µL aliquots of each were pooled for genomic DNA isolation and whole-genome sequencing at the Center for Genomics and Bioinformatics at Indiana University Bloomington. Geneious (Biomatters) was used to map single-end 150 bp reads to the *V. vulnificus* reference genome (105) to identify Transposon (Tn) insertion sites.

### Construction and complementation of the Δ*plzD* strain

For markerless deletion of *plzD*, PCR fragments corresponding to 1 kb upstream (primers DplzD-pRE112-SP-F and DplzD-SP-1) and downstream (primers DplzD-SP-2 and DplzD-pRE112-SP-R) of the gene were amplified, fused by overlapping PCR, and cloned into the multiple cloning site (MCS) of pRE112 (106) by Gibson assembly. Colonies were selected on LB Cm plates, and plasmids containing the correct insert were identified by PCR. Following conjugation to *V. vulnificus*, cointegrates were selected on LB Col Cm plates and then gridded onto plates that lacked Cm but contained 20% sucrose for *sacB*-mediated counterselection. The mutants were white on LB X-Gal plates and confirmed by PCR. For complementation, *plzD* was amplified with primers plzD-pC2X6HIST-SP-F and plzD-pC2X6HIST-SP-R and cloned downstream of the *P_tac_* promoter in pC2X6HT (84). This plasmid was then used to introduce the *plzD^R140A^* mutation by inverse PCR with primers 898PlzD-R140A-F and 898plzD-SPM-R. The plasmid was sequenced to confirm nothing else was altered. These plasmids were conjugated to Δ*plzD* cells and expression was induced with 10 µM IPTG.

An in-frame deletion of the gene encoding the flagellar motor protein FliG was accomplished as follows: PCR fragments that included 1 kb upstream to the first 9 bp of *fliG* (primers DfliG-1 and DfliG-TpK7-2) and the last 9 bp to 1 kb downstream of *fliG* (primers DfliG-TpK7-3 and DfliG-4) were amplified and fused to a central trimethoprim antibiotic resistance cassette (primers TmR-F and TmR-R). The primers included complementary 35 bp overhangs so that the PCR products could be fused together using the Gibson assembly kit (New England BioLabs) to replace *fliG* with a trimethoprim marker. *V. vulnificus* strains carrying the pMMB-TfoX expression plasmid (107) were induced overnight in LB medium containing Ap and IPTG at 30°C. A 10 µL aliquot of cells was added to 500 µL of IO-20 containing 100 µM IPTG and the Gibson reaction mixture. The transformation mixture was incubated statically overnight at 30°C. The next day, 1 mL of LB medium was added to the tube, and the cells were allowed to outgrow for 3 h before plating on LB Tp plates. Allelic replacement of the target region was confirmed by PCR. This method was also used to delete the *fliM* (motor), *motA* and *motB* (stator), or *cheY3* (switch) genes in the Δ*plzD* background. See Table S1 for primer sequences.

### Construction of the *P_cat_plzD* strain for infection studies

The 160 bp *P_cat_* promoter of pSW23T (108) (amplified with primers PcatFor and PcatRev) was fused to *plzD* (amplified with primers PcatplzD-F and PcatplzD-R) by PCR for ectopic integration into the chromosomal β-galactosidase (*lacZ*) gene. This product was flanked by 1 Kb regions (amplified with VvlacZ-1 and VvlacZ-2 and VvlacZ-3 and VvlacZ-4, respectively) of *lacZ* using the Gibson ligation kit (NEB). The reaction was transformed into the Δ*plzD* strain carrying pMMB-TfoX as described above. Transformants were plated on media including Xgal, and colonies that were white on LB X-Gal plates were confirmed by PCR. Since *P_cat_plzD* insertion inactivated *lacZ*, a strain in which the *lacZ* promoter (*P_lacZ_*) was deleted was used as a control. To delete *P_lacZ_*, PCR fragments corresponding to 1 kb upstream (primers DPlacZ-pRE118-SP-F and DPlacZ-SP-1) and downstream (primers DPlacZ-SP-2 and PlacZ-pRE118-SP-R) of the lacZ promoter region were amplified, fused, and cloned into the MCS of pRE118 (106) by Gibson assembly. Colonies were selected on LB Cm plates, and plasmids containing the correct insert were identified by PCR. Following conjugation to *V. vulnificus*, cointegrates were selected on LB Col Cm plates and then gridded onto plates that lacked Cm but contained 20% sucrose for *sacB*-mediated counterselection. Promoter deletion mutants were white on LB X-Gal plates and confirmed by PCR.

### Protein purification and micro-dialysis assay for c-di-GMP binding

Expression of His-tagged PlzD and PlzD^R140A^ from pC2X6HT was induced in *E. coli* BL21(DE3) cells (Novagen) with 100 µM IPTG. The proteins were purified by His-Trap Ni-NTA affinity chromatography as previously described (84). To exchange buffer, proteins were applied to a 5 mL HiTrap desalting column and eluted with 10 mM Tris (pH 7.5) containing 100 mM NaCl and 5 mM MgCl_2_. Proteins were used immediately or stored at 4°C for up to 2 weeks. For longer-term storage, glycerol was added to 50% and the samples were kept at −20°C. The concentration of the purified proteins was determined by Nanodrop A280 nm measurement.

c-di-GMP binding assays were done by micro-equilibrium dialysis (89) using a device with 2 µL × 25 µL chambers separated by a re-generated cellulose membrane with 10 KDa cut-off (Harvard Apparatus). Twenty-five microliters of c-di-GMP (10 µM) and 25 µL of increasing concentrations of His-tagged PlzD or PlzD^R140A^ (2.5, 5, 10, 20, and 40 µM) were added to opposing chambers of the device. Buffer alone was used for no-protein controls. The assembled device was gently rocked at 4°C overnight to reach equilibrium. The c-di-GMP concentration in the ligand chamber was determined by UV absorbance (253 nm, ε = 28,600 M⁻¹ cm⁻¹) at room temperature (109) using a Synergy H1 microplate reader with Take3 accessory (Biotek). The absorbance of unbound c-di-GMP in the ligand chamber was plotted versus the protein:ligand ratio in Prism 9. Overnight dialysis for the no-protein control showed that the absorption spectra of samples from both chambers were equal, indicating that overnight incubation was sufficient to reach equilibration and that c-di-GMP does not interact with the membrane or chamber walls.

### Fluorescence microscopy

The pC2X6HT plasmid expressing the *plzD-gfp* fusion was conjugated into the indicated *V. vulnificus* strains and expression was induced with IPTG (10 µM). Images of individual cells were captured after cells were mounted on low melting point agarose pads or using the CellAsic microfluidic perfusion system (ONIX) with integrated temperature controller and B04A bacterial microfluidic plates (EMD Millipore, Billerica, MA). The CellAsic ONIX FG software (v 5.0.2.0) was used to control flow rate and deliver fresh medium. The chambers were loaded by perfusion of 50 µL of a 10^6^ cells per milliliter bacterial suspension at 2 lb/in^2^ for 15 s to prime the system followed by 4 lb/in^2^ for 15 s to trap the cells. The chamber was then rinsed at 1 lb/in^2^ for 30 s followed by 2 lb/in^2^ for the remainder of the assay. Single-cell static, time-lapse images and movies were captured on an Olympus IX83 inverted microscope using a 100×, 1.3 numerical aperture phase contrast objective. Phase contrast and fluorescence images were obtained with a Hamamatsu ORCA-R2 digital charge-coupled device camera, and the light source was the Xcite 120 light-emitting diode (Lumen Dynamics, Mississauga, ON, Canada). Emission filters were purchased from Chroma Technology (Bellows Falls, VT). The specific emission filters were DAPI-5060C-OMF [excitation (EX) filter, 377/50 nm; emission (EM) filter, 447/60 nm; dichroic mirror (DM), 409 nm], GFP-3035D-OMF (EX filter, 473/31 nm; EM filter, 520/35 nm; DM, 495 nm), and mCherry-B-OFF (EX filter, 562/40 nm; EM filter, 641/75 nm; DM, 593 nm). Data from three biological replicates were analyzed for each strain. Images were processed using cellSense software (Olympus) and those presented are from a single representative experiment.

### Aggregation and biofilm assays

Strains expressing *gfp* were grown in LB, LB plus Ca^2+^ (15 mM), IO-20, or IO-35 media overnight at 30°C. The cells were passed over a 50 µm filter, and flow cytometry of the filtrates was performed on a FACSCalibur flow cytometer (BD Biosciences) equipped with a blue laser (λex = 488 nm) and a band pass filter measuring green fluorescence (FL1; 530/30 nm). The sample flow rate was set at “low” (12 ± 3 µL/min) and 70,000 events were recorded within a preset gate defining the viable cell population. Analyses were performed on biological triplicates and the data were analyzed using FlowJo X (Tree Star).

Polydimethylsiloxane glass flow cell devices containing eight 40 × 5 × 1 mm^3^ chambers were fabricated as previously described (110). The chambers were sterilized by sequential treatment with 50 mL each of 3% H_2_O_2_, sterile H_2_O, and IO-20 prior to inoculation. Mid-log phase cells for each strain (OD_600_ of 0.1) were seeded into separate flow cell chambers. Control chambers for background fluorescence contained medium only. Initial attachment (no flow) proceeded for 20 min, followed by a flow rate of 0.25 mL/min in IO-20 tryptone for 16 h. The chambers were then exposed to the same medium containing 2.5 µM Hoechst stain (ThermoFisher) for 10 min. Biofilm images and z-stacks (20 × 1 µm slices) were captured with an Olympus IX83 microscope using a UPLSAPO 40× silicon oil immersion objective [numerical aperture (NA), 1.25; working distance (WD), 0.3 mm]. Background fluorescence was subtracted from each sample, and quantitative analysis to determine biomass was performed using cellSense (Olympus) and Comstat (111). Data from three biological replicates were analyzed for each strain. The images presented are from a single representative experiment.

### 3D motility tracking

*V. vulnificus* was grown overnight in IO-20 tryptone Col media at 30°C. An aliquot was gently washed once in IO-20 medium (5 min at 4,000 g) and then diluted to an OD_600_ of 0.1 in fresh IO-20 medium. Tracking chambers were made by layering three parafilm frames between a microscope slide and a 22 mm × 22 mm no. 1.5 coverslip. The chamber was sealed by placing it in a 55°C dry oven for 10 min. A culture of bacteria grown overnight was diluted into IO-20 medium to an OD_600_ of 0.1 and flowed into the chamber at one edge by capillary action. The edges of the unit were sealed with hot VALAP, and the samples were immediately imaged on the above-mentioned microscope equipped with an Olympus long working distance (LWD) air condenser (NA, 0.55) and an Olympus LUCPLFLN LWD 40× phase contrast objective (Ph2; NA, 0.6). The correction collar was set to 1.2. For each movie recording, frames were acquired at a rate of 15 Hz and an exposure time of 5 ms with a Hamamatsu Orca-R2 camera (1,344 by 1,024 pixels). The objective was positioned so that the focus was 50 µm above the coverslip. The data were saved as a stack of 16-bit tiff files of 300 images each. Image stacks were background corrected and aligned to a 3D position reference library in Matlab using a custom-written tracking program to analyze motility behavior (59, 112). Each re-orientation event (flicks and reversals) identified by the program was manually confirmed based on the angle and swimming speed between the corresponding vectors for at least 100 trajectories prior to statistical analysis. Trajectories with an average speed of less than 10 µm/s or with a duration of less than 50 frames were ignored. Plots were created using data from three stacks of three biological replicates for each strain. Traces from a single representative experiment are shown.

### Structural modeling

The crystal structure of PlzD (PDB 2RDE) of *Vibrio cholerae* was used as a scaffold to build structural models for PlzD using AlphaFold2 (113). Chimera (114) was used for molecular visualization. Five homology models were generated for each protein, and the model with the best overall agreement score was analyzed further.

We elected to internally tag PlzD with superfolder GFP (^sf^GFP) since soft agar motility assays and Western blotting indicated that amino or carboxy terminal tags were inactive or unstable (data not shown). Because the structure of PlzD is not known, we first compared the known structures of PlzD and the *Bacillus subtilis* YcgR homolog DgrA, in which a stably tagged functional fusion was made by inserting ^sf^GFP into an exposed N-terminal loop (115). Structural modeling revealed a homologous N-terminal exposed loop (between P^50^ and V^51^) and two C-terminal exposed loops (between N^152^ and D^153^, and between P^174^ and M^175^) in PlzD that were predicted to be conserved in PlzD as potential targets for ^sf^GFP insertion that would minimally interfere with function as a monomer or dimer. The C1 loop fusion (PlzD^sfGFP^) was stably expressed in *V. vulnificus* and inhibited motility as well as native PlzD (Fig. S4).

### Oyster colonization assay

Overnight, ice-packed shipments of live Eastern oysters (*Crassostrea virginica*) were acclimated at 20°C for 1 h and thoroughly scrubbed to eliminate epifauna. Sets of five similarly sized oysters were placed in open autoclavable polypropylene cages and transferred to a custom maintenance system consisting of 40-gallon aquaria filled with IO-20. Each tank was equipped with two CNZ Wavemaker 1320-GPH circulators and a HOB-XC protein skimmer (AquaMaxx) with a Cobalt 12–16 skimmer pump. Aquaria were maintained a constant temperature of 20°C with a HAAKE DC10/EK20 immersion cooler system (ThermoFisher Scientific). Oysters were fed Tetraselmis 3600 and Shellfish Diet 1800 (Reed Mariculture) for at least 48 h prior to experimentation and they could be readily maintained for a month. Oysters were rinsed and transferred to 5-gallon aquaria containing 8 L of IO-20 at 20°C for depuration with a combination of Cm and Tc for 16 h to lower endogenous bacterial loads and promote establishment of the test inoculum. Oysters were then transferred to 5-gallon aquaria equipped with an activated charcoal filtration cartridge and 8 L of fresh IO-20 for overnight incubation to eliminate residual antibiotic from oyster tissues. Depurated oysters were then transferred to individual 5-gallon aquaria with 8 L of fresh IO-20 for inoculation with *V. vulnificus*. Bacteria were grown overnight at 20°C in IO-20 supplemented with 1% tryptone. Inoculums were added to the aquaria at a final concentration of 10^6^ CFU/mL for natural uptake via filter feeding. Control oysters did not receive bacteria. Oysters were shucked 36 h after inoculation under sterile conditions using an oyster knife that was rinsed with 70% ethanol and flamed. Oyster meats from sets of five animals were collected and rinsed with 100 mL of sterile Vibrio-specific phosphate-buffered saline (VPBS) to remove planktonic cells and loosely attached bacteria. Sterile VPBS (200 mL) was added to the rinsed meat and the sample was homogenized using a Magic Bullet Blender (Capbran) in three pulses of 20 s each. The homogenates were serially diluted in VPBS and CFU counts were determined on LB Col plates following overnight incubation at 30°C. No isolates were ever recovered on LB Col plates for homogenates of uninoculated control oysters. Experiments were performed in triplicate for each strain.

### Mouse survival and skin wound assays

Strains were inoculated from single colonies into IO-20 tryptone and were cultured overnight at 30°C. The overnight culture was seeded at a 1:100 dilution into fresh media and incubated at 30°C until the bacteria reached an OD_600_ of 1.0. Bacteria were collected by centrifugation and washed once with phosphate-buffered saline (PBS). Bacterial cell concentrations were confirmed by plate counts. Five-week-old female CD-1 mice housed under pathogen-free conditions were pretreated with an intraperitoneal injection of iron dextran (250 µg of iron-dextran per gram of body weight) 60 min before inoculation with the bacterial strains. Groups of mice (*n* = 5) were injected intraperitoneally with 0.1 mL of 10^7^ CFU of each bacterial strain. Mice were observed for up to 120 h post-infection with the recording of deaths or moribund animals.

For excisional skin wounds, mice were anesthetized with 5% isoflurane in 100% O_2_ and subsequently maintained using 3% isoflurane. The dorsal skin was shaved to remove hair, thoroughly cleaned, and disinfected prior the surgery using betadine scrub and 75% EtOH solution. Preoperative analgesia was applied using a subcutaneous injection of Meloxicam (2 mg/kg) and a full-thickness 10 mm wound (through the panniculus carnosus layer) was created between the shoulder blades of the mice using a scalpel. A silicone ring splint (0.5 mm thick with a 20 mm inner diameter, Life Technologies) centered around the wound was affixed to the skin with immediate‐bonding adhesive (Gluture) and nylon sutures to prevent wound contraction. 10^7^ CFU of the indicated bacterial strain was applied to the wound site and then it was covered with Tegaderm semi-occlusive dressing (3M). Wounds were digitally photographed 8 h post-infection. Experiments were performed in triplicate for each strain. The images presented are from a single representative experiment. Mice were purchased from Jackson Laboratory.

## SUPPLEMENTAL MATERIAL

Figure S1 mbio.01536-23-s0001.tif PlzD-mediates inhibition of *V. vulnificus* motility by elevated cellular c-di-GMP.

Figure S2 mbio.01536-23-s0002.tif Structural alignment of the PilZ domains of PlzD from *V. cholerae* and *V. vulnificus*.

Figure S3 mbio.01536-23-s0003.tif The PlzDsfGFP fusion is stable and functional.

Figure S4 mbio.01536-23-s0004.tif Polar localization of PlzD in planktonic and biofilm cells.

Figure S5 mbio.01536-23-s0005.tif Growth of *V. vulnificus* strains in IO20.

Figure S6 mbio.01536-23-s0006.tif Virulence of the wild-type and △*plzD* strains.

Legends mbio.01536-23-s0007.docx Supplemental figure legends.

Table S1 mbio.01536-23-s0008.docx Primers used in this study.

ASM does not own the copyrights to Supplemental Material that may be linked to, or accessed through, an article. The authors have granted ASM a non-exclusive, world-wide license to publish the Supplemental Material files. Please contact the corresponding author directly for reuse.
