# Supplementary figures and images for: PlzD modifies Vibrio vulnificus foraging behavior and virulence in response to elevated c-di-GMP
Source: mBio. 2023 Oct 6;14(5):e01536-23. doi: 10.1128/mbio.01536-23 (PMC10653909; doi:10.1128/mbio.01536-23)

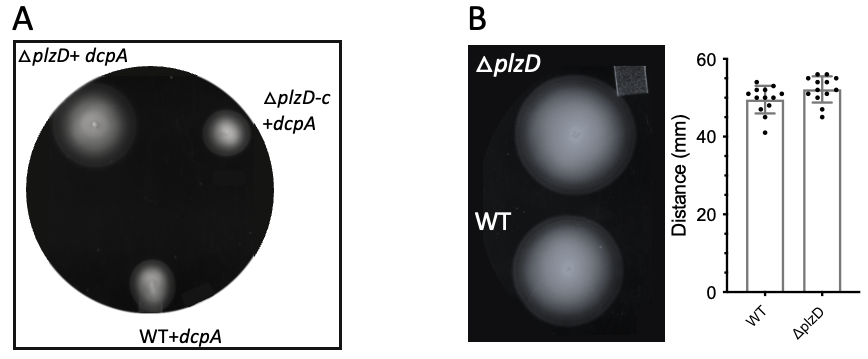

Supplement: Figure S1 — PlzD-mediates inhibition of V. vulnificus motility by elevated cellular c-di-GMP. [file mbio.01536-23-s0001.tif]

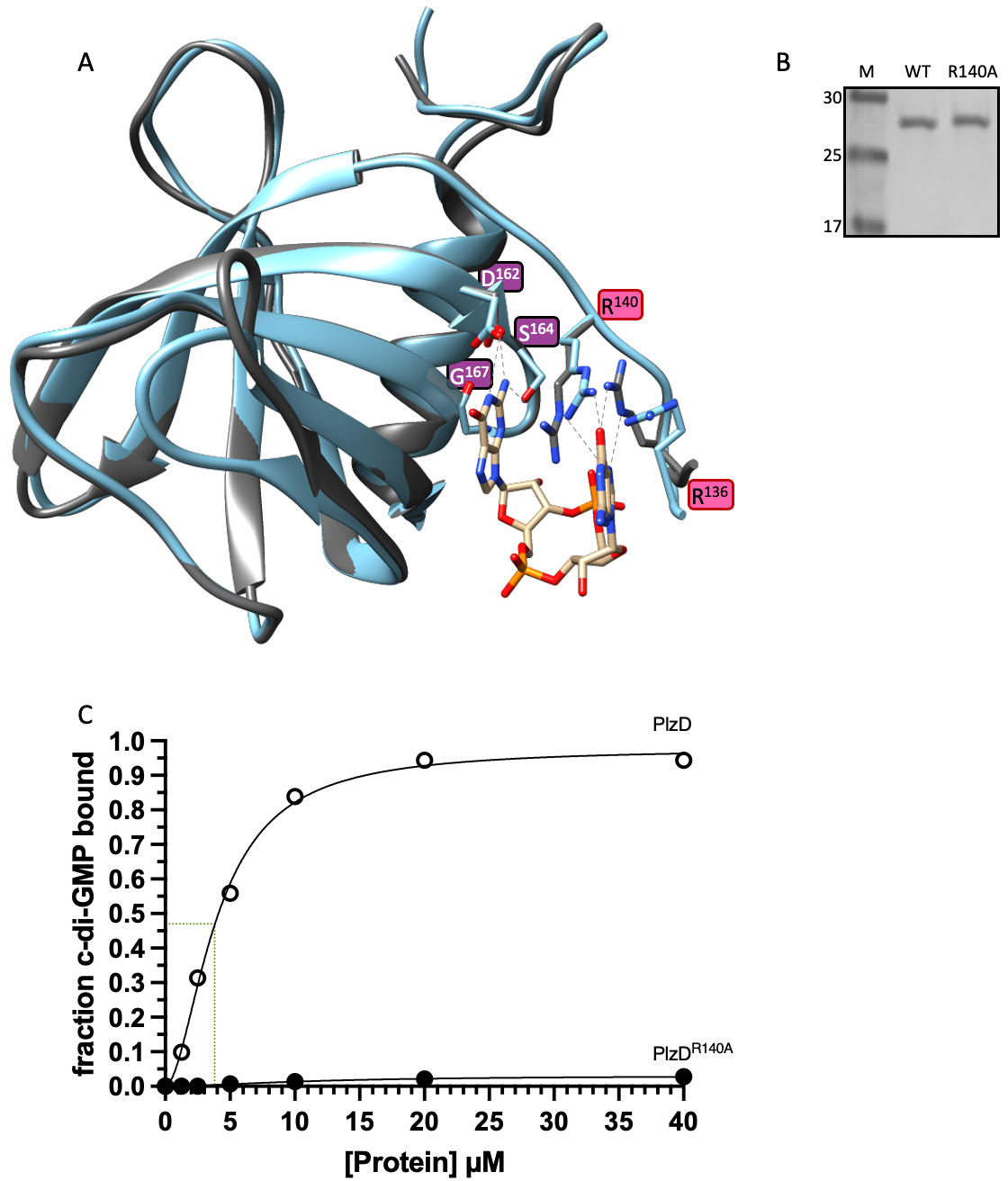

Supplement: Figure S2 — Structural alignment of the PilZ domains of PlzD from V. cholerae and V. vulnificus. [file mbio.01536-23-s0002.tif]

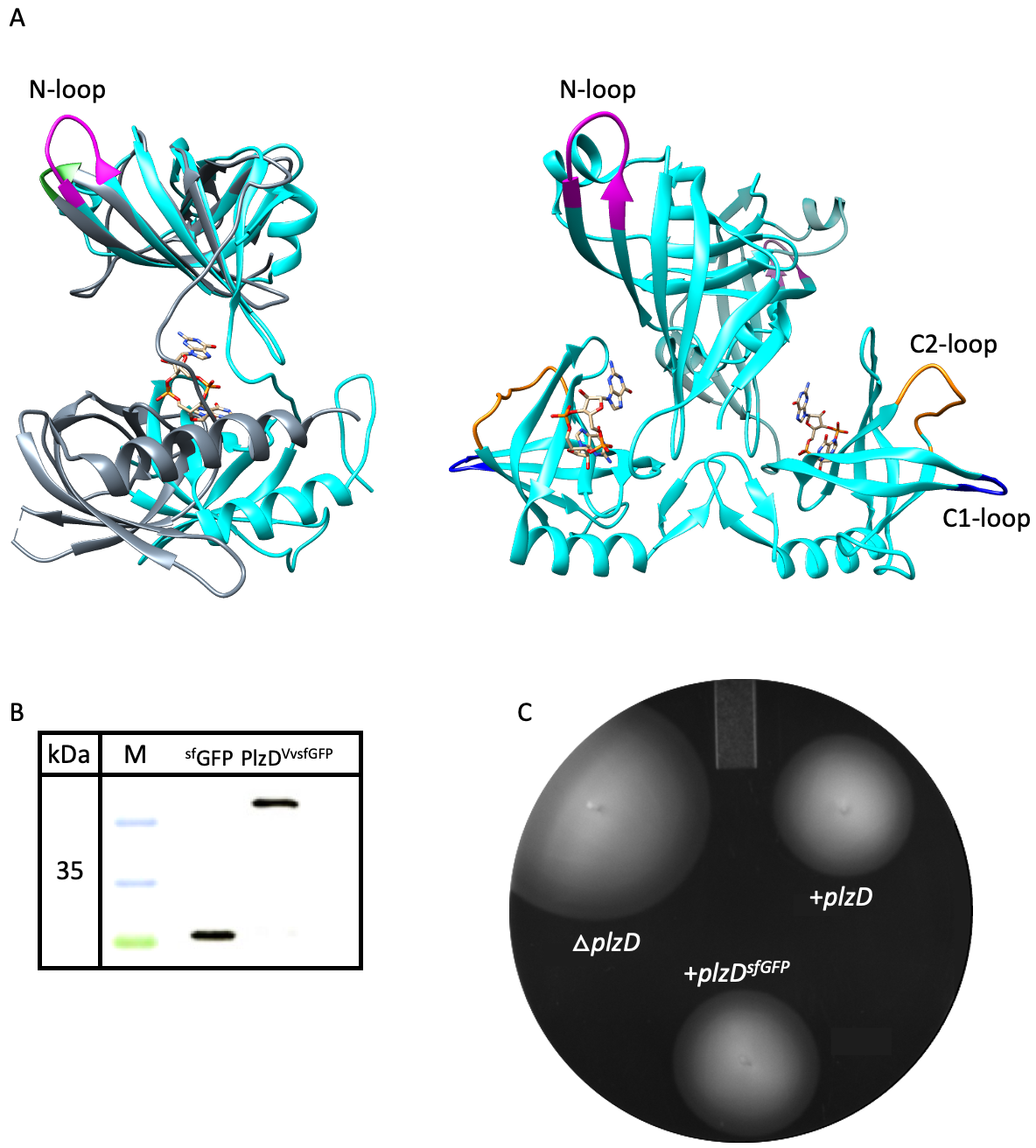

Supplement: Figure S3 — The PlzDsfGFP fusion is stable and functional. [file mbio.01536-23-s0003.tif]

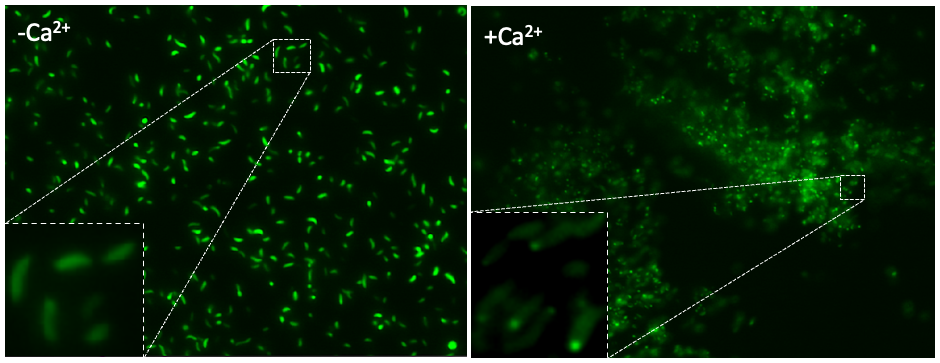

Supplement: Figure S4 — Polar localization of PlzD in planktonic and biofilm cells. [file mbio.01536-23-s0004.tif]

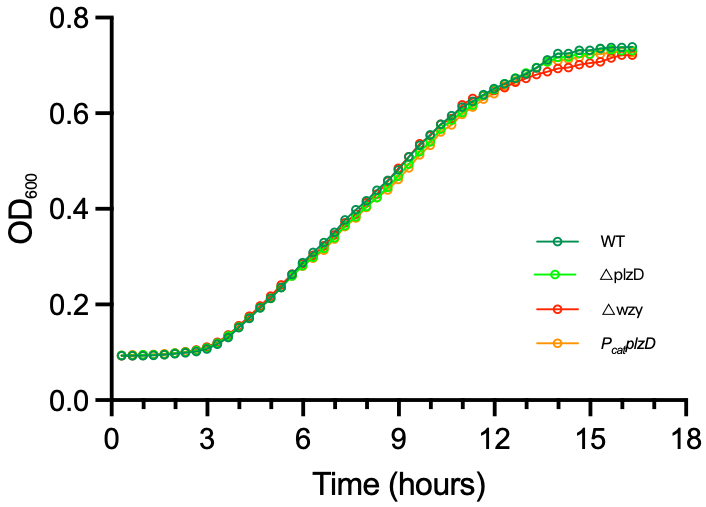

Supplement: Figure S5 — Growth of V. vulnificus strains in IO20. [file mbio.01536-23-s0005.tif]

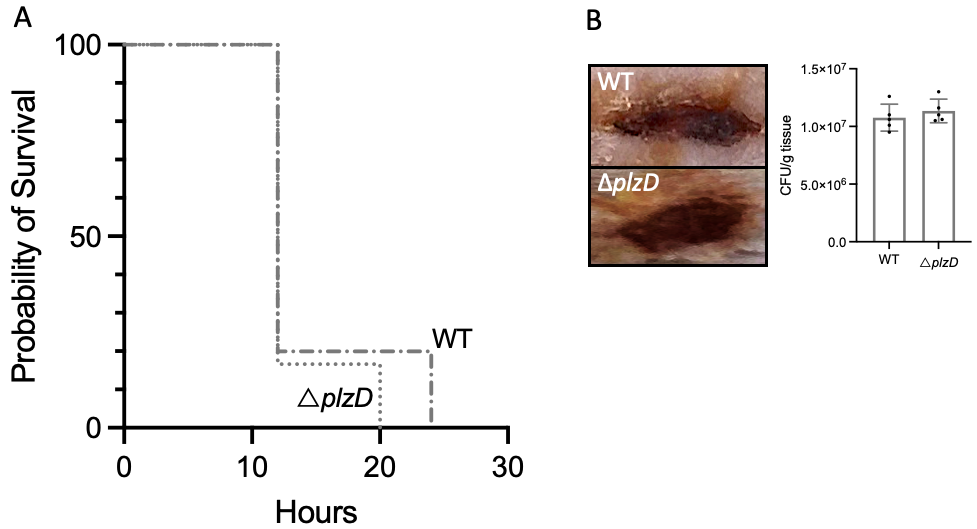

Supplement: Figure S6 — Virulence of the wild-type and △plzD strains. [file mbio.01536-23-s0006.tif]
