# Supplementary material for: PlzD modifies Vibrio vulnificus foraging behavior and virulence in response to elevated c-di-GMP
Source: mBio. 2023 Oct 6;14(5):e01536-23. doi: 10.1128/mbio.01536-23 (PMC10653909; doi:10.1128/mbio.01536-23)
Supplement: Legends — Supplemental figure legends. [file mbio.01536-23-s0007.docx]

**Supplemental Figure Legends**

**Figure S1. PlzD-mediates inhibition of *V. vulnificus* motility by elevated cellular c-di-GMP. A,** motility of wildtype (WT), △*plzD* and complemented (-c) cells expressing *dcpA*. B, Motility of the WT and △*plzD* strains. To the right is a plot of the corresponding motility zones for the strains in B. It shows the respective mean values and error bars represent the standard deviation of multiple assays. No statistically significant difference was found (unpaired Student's *t*-test (two-tailed), p value, <0.05).

**Figure S2. Structural alignment of the PilZ domains of PlzD from *V. cholerae* and *V. vulnificus*.** A, overlay of the RXXXR and D/NXSXXG motifs (stick configuration) of *V. vulnificus* PlzD (cyan ribbon) with the same motifs in the known c-di-GMP binding site of *V. cholerae* PlzD (grey ribbon, PDB code 2RDE). The RxxxR motif is boxed in pink and the D/NxSxxG motif is boxed in purple. Nitrogen, oxygen, and phosphorus atoms of c-di-GMP (shown in stick configuration) are colored blue, red, and orange, respectively. B, Western blot of equal amounts of total protein from Δ*plzD* cells expressing either N-terminally His-tagged PlzD or PlzD^R140A^ that was detected with an anti-6xHis antibody. M, protein marker (kDa). C, Plot of the fraction of free c-di-GMP bound versus the concentration of PlzD and PlzD^R140A^ following micro-equilibrium dialysis. The dotted line is the concentration at which half the c-di-GMP was bound by PlzD molecules (K_D_).

**Figure S3. The PlzD^sfGFP^ fusion is stable and functional.** A, on the left is a structural alignment of *V. cholerae* PlzD (2RDE, cyan) and the *B. subtilis* YcgR homolog DgrA (grey), into which sfGFP was stably inserted into an N-terminal loop (N-loop; region in green). The homologous region is highlighted in magenta in the PlzD monomer and dimer (structure on the right). Two C-terminal loops (C1 in blue and C2 in orange) were also identified as possible sfGFP insertion sites. B, Western blot of equal amounts of total protein from Δ*plzD* cells expressing *plzD* or *plzD^sfgfp^*. The His-tagged proteins were detected with an anti-His antibody. M, protein marker (kDa). C, motility of the Δ*plzD* strain carrying the empty vector or expressing *plzD* or the *plzD^sfgfp^* fusion (+).

**Figure S4. Polar localization of PlzD in planktonic and biofilm cells.** The distribution of the PlzD^sfGFP^ in planktonic (left) and biofilm (right) *V. vulnificus* ∆*plzD* cells (40X magnification). Biofilm formation was stimulated by adding Ca^2+^ (10 mM final concentration) to the LB media. Insets show the magnified view of individual cells from the indicated region (white dashed box).

**Figure S5.** **Growth of *V. vulnificus* strains in IO20.** Growth of the wildtype (WT), ∆*wzy,* ∆*plzD* and *P_cat_plzD* strains at 30°C in IO20 supplemented with 1% peptone. No statistically significant difference in growth between the strains was found (one-way ANOVA, Kruskal-Wallis test with Dunn’s multiple comparisons post hoc test, p value, <0.05). Error bars were omitted for easier viewing.

**Figure S6.** **Virulence of the wildtype and △*plzD* strains.** A, survival curve for mice injected with the wildtype strain (WT) or the ∆*plzD* strain. No statistically significant difference between the strains was found (Mantel-Cox test). B, representative images of wounds 8-hr post-infection with the same strains. To the right is a plot of the CFU recovered per gram of tissue from each wound site. No statistically significant difference was found (unpaired Student's *t*-test (two-tailed), p value, <0.05).
