# Supplementary material for: PlzD modifies Vibrio vulnificus foraging behavior and virulence in response to elevated c-di-GMP
Source: mBio. 2023 Oct 6;14(5):e01536-23. doi: 10.1128/mbio.01536-23 (PMC10653909; doi:10.1128/mbio.01536-23)
Supplement: Table S1 — Primers used in this study. [file mbio.01536-23-s0008.docx]

Table S1. Primers used in this study.

| **Name** | **Sequence (5’ to 3’)** |
| --- | --- |
| DplzD-pRE112-SP-F | GTACCGGGTTGAGAAGCGGTGTAAGTGAACTGCATCTATTCGCCCAACGTCAATTG |
| DplzD-SP-1 | TCACTTATCGGCACGCCTACATTGTTCCTTATCCTAATGAAA |
| DplzD-SP-2 | TTTCATTAGGATAAGGAACAATGTAGGCGTGCCGATAAGTGA |
| DplzD-pRE112-SP-R | TTGCTACGCCTGAATAAGTGATAGGGCCCGATCCCTCTGTTCGTGCAGACGTCG |
| plzD-pC2X6HIST-SP-F | AAGGACCATAGCATATGCATCATCACCATCACCACATGAATTTACCTATGGCTAAACT |
| plzD-pC2X6HIST-SP-R | GTGCCAAGCTTGCCTGCAGGTCGACTCTAGAGGATCTAATGTTTGAGTTTGAGCTTG |
| 898PlzD-R140A-F | GAGACAGCGTACGAGGTTAACCTCCCAGCGAAAGCTTA |
| 898plzD-SPM-R | AACCTCGTACGCTGTCTCTTTGCGAAGTTGAGCCACTTG |
| DfliG-1 | CGCTTGCTCAATTCTGGTTC |
| DfliG-TpK7-2 | GTCGACGGATCCCCGGAATGTTAGCCATTTACCATCCAATT |
| DfliG-TpK7-3 | GAAGCAGCTCCAGCCTACATTCTTGTAATAACAGACAAGCC |
| DfliG-4 | CCAGCGTTAAACCGACAAC |
| DfliM-1 | AATGTCGCAGCGCAACAACC |
| DfliM-TpK7-2 | GTCGACGGATCCCCGGAATATCGGTCACGCTATACCTACT |
| DfliM-TpK7-3 | GAAGCAGCTCCAGCCTACAGATGATTAATGCTCGCGACTG |
| DfliM-4 | AACGCGGTCTCTTCCGCTT |
| DmotAB-1 | GTGAACGCGAGTGTTTGTA |
| DmotAB-TpK7-2 | GTCGACGGATCCCCGGAATTAAATCCACAAAGCACTCCTCATGCTATTTTC |
| DmotAB-TpK7-3 | GAAGCAGCTCCAGCCTACAGAGCAGTAATTGGGTACGTGAGTTGAAT |
| DmotAB-4 | CTAAAGCTTGAATATCAAACTTC |
| Dchey3-1 | TGTGAGCCTCGAACTCGTA |
| DcheY3-TpK7-2 | GTCGACGGATCCCCGGAATTTTATTCAAAATTGCCTCCACTGAATG |
| DcheY3-TpK7-3 | GAAGCAGCTCCAGCCTACACGTTTATAAACCTCGTTTGAAGATTACTT |
| Dchey3-4 | ATTGAGAAGATCCCTGTCTTC |
| DPlacZ-pRE118-SP-F | GTACCGGGTTGAGAAGCGGTGTAAGTGAACTGCATCTCATGCCATTCAAACCACAA |
| DPlacZ-SP-1 | TCGGCCTGCGTGAACTGGTGGTGATGCGTGTGAGTGC |
| DPlacZ-SP-2 | GCACTCACACGCATCACCACCAGTTCACGCAGGCCGA |
| DPlacZ-pRE118-SP-R | ACGTGGCTTTCCCCCCCCCCCCTGCAGGTGATCCCTCACACAGTTCGTACCAACG |
| PcatFor | TGGTGTCCCTGTTGATACCG |
| PcatRev | TTTAGCTTCCTTAGCTCCTGAA |
| PcatplzD-F | TTCAGGAGCTAAGGAAGCTAAAATGAATTTACCTATGGCTAAACT |
| PcatplzD-R | CTAATGTTTGAGTTTGAGCTTG |
| VvlacZ-1 | ATGTGTTCATCACGCCGGAG |
| VvlacZ-2 | CGGTATCAACAGGGACACCAAGCGTACATCGGCGCAATG |
| VvlacZ-3 | CAAGCTCAAACTCAAACATTAGCGCGTGAATACCCTTGTTGC |
| VvlacZ-4 | GCGTTTCGCTCTCGCAATGA |
